# InterMiG: international differences in the therapeutic approach to migraine patients in specialized headache centers

**DOI:** 10.1186/s10194-021-01258-y

**Published:** 2021-05-24

**Authors:** AB Gago-Veiga, J-I Huhn, N Latysheva, A Vieira Campos, M Torres-Ferrus, A Alpuente Ruiz, S Sacco, I Frattale, R Ornello, R Ruscheweyh, IB Marques, A Gryglas-Dworak, C Stark, VJ Gallardo, P Pozo-Rosich

**Affiliations:** 1 Headache Unit, Department of Neurology, La Princesa Research Institute. Hospital Universitario de la Princesa, Madrid, Spain; 2 Praxis Gendolla. Zentrum für Neurologie und Schmerztherapie, Essen, Germany; 3 Department of Neurology, Institute for Professional Education, I.M.Sechenov First Moscow State Medical University (Sechenov University), Moscow, Russia; 4 Headache Unit, Neurology Department, Hospital Universitari Vall d’Hebron, Barcelona, Spain; 5 Headache and Neurological Pain Research Group, Vall d’Hebron Research Institute, Departament de Medicina, Universitat Autònoma de Barcelona, Barcelona, Spain; 6 Neuroscience Section, Department of Applied Clinical Sciences and Biotechnology, University of L’Aquila, L´Aquila, Italy; 7 Department of Neurology, Ludwig Maximilians University Munich, Munich, Germany; 8 Department of Neurology, Hospital da Luz Lisboa, Lisbon, Portugal; 9 Private Headache Clinic, Wroclaw, Poland; 10 Austin Health, Heidelberg, Australia

**Keywords:** Migraine, Preventive treatment, International, Differences, Antidepressant, Antiepileptic, Beta-blockers, OnabotulinumtoxinA

## Abstract

**Background:**

There is currently a wide therapeutic arsenal for migraine patients, without a single first-line preventive drug and we choose the different available alternatives taking into account comorbidities, national guidelines, previous treatments and personal experiences.

Our objective was to evaluate the differences in the use of migraine treatments between neurologists from different countries.

**Methods:**

This is a multi-centre observational study carried out by neurologists from specialized headache units in seven countries, retrospective with consecutive inclusion of all patients presenting with a migraine diagnosis, over a period of three months.

**Results:**

A total of 734 patients were recruited but only 600 were considered in the analysis in order to homogenize the patient cohorts from countries: 200 Spain (ES), 100 Italy (IT), 85 Russia (RUS), 80 Germany (DE), 60 Portugal (PT), 45 Poland (PL) and 30 Australia (AU). 85.4 % of patients were women with a mean age of 42.6 ± 11.8 years. Considering previous and current preventive treatment, the order of use was: antidepressants (69.3 %), antiepileptic drugs (54.7 %), beta-blockers and antihypertensive drugs (49.7 %), OnabotulinumtoxinA (44.0 %) and others (36.2 %).

Statistically significant differences were found between all pharmacological classes: antidepressants were commonly used in all countries, with the exception of Poland (AU: 76.7 %, IT: 71.0 %, DE: 60.0 %, PL: 31.1 %, PT: 71.7 %, RUS: 70.6 %, ES: 78.5 %; *p* < 0.0001); antiepileptic drugs were more frequently prescribed in Portugal, Australia and Spain (AU: 73.3 %, IT: 40.0 %, DE: 37.5 %, PL: 48.9 %, PT: 85.0 %, RUS: 29.4 % and ES: 69.0 %; *p* < 0.0001); beta-blockers and antihypertensive drugs were frequently used in all countries except Italy (AU: 60.0 %, IT: 14.0 %, DE: 53.8 %, PL: 48.9 %, PT: 68.3 %, RUS: 49.4 % and ES: 59.0 %; *p* < 0.0001); BTX-A were predominately used in Spain, Italy and Australia (AU:56.7 %, IT:58.0 %, DE:20.0 %, PL: 42.2 %, PT: 26.7 %, RUS: 24.7 % and ES: 58.5 %; *p* < 0.0001) and others were most frequently used in Poland (AU: 0.0 %, IT: 19.0 %, DE: 42.5 %, PL: 95.6 %, PT: 31.7 %, RUS: 3.5 % and ES: 49.5 %; *p* < 0.0001). If only patients without comorbidities are considered (200/600), statistically differences between countries persist in all preventive treatments.

**Conclusions:**

There is heterogeneity in the choice of preventive treatment between different countries. Prospective comparative studies of the different oral and subcutaneous alternatives would help to create a global therapeutic algorithm that would guarantee the best option for our patients.

## Background

Migraine is a chronic disease accounting for a large proportion of disability across all ages, especially in the young. In fact, in 2019 migraine was named the first was named the first disabler under the age of 50 [1].

It has been estimated that up to 38 % of migraine patients need preventive treatment [2]. However, the international guidelines on migraine prophylaxis only contain a list of medications, which have undergone randomized clinical trials and show their level of evidence [3, 4]. These documents do not give recommendations on the choice of first-line drugs.

More recent guidelines have summed up the real-life experience of general practitioners and headache centers and recommend that the choice of a migraine prophylactic drug should be based on the attack frequency (episodic vs. chronic), comorbid diseases and the patient’s individual needs [5, 6]. Differences in national healthcare systems and reimbursement policies also factor in the choice of preventive strategies in different countries.

This heterogeneity of treatment practices may also be partly explained by the differences in headache care systems. Whereas in some countries general practitioners successfully treat most patients with migraine, in others countries this is not the case, resulting in a large number of drug-naïve patients referred to third-level headache centers. Such differences in patient populations and proportions of refractory headaches seen by specialized centers may also influence drug choice.

In consequence, the objective of this study was to evaluate the differences of preferences of use of acute and preventive migraine treatments internationally across headache centers in several countries.

### Methods

### Study population

We performed a multicentre retrospective observational study with consecutive inclusion of patients, carried out by neurologists from specialized headache units in seven countries: Spain (ES) (La Princesa University Hospital and Vall d´Hebron University Hospital), Germany (DE) (Praxis Gendolla and Ludwig Maximilians University Munich), Russia (RUS) (Sechenov University), Italy (IT) (University of L´Aquila), Portugal (PT) (Hospital da Luz Lisboa), Poland (PL) (Private Headache Clinic Wroclaw) and Australia (AU) (Austin Health). Each centre was required to contribute at least 30 patients, starting data collection in February 2019 and continuing for a maximum of three months. The inclusion criteria were: patients with migraine diagnosed according to the International Classification of Headache Disorders-3 [7], who were 18 years of age, with no upper age limit. Patients were excluded when not enough data was collected in their medical history.

### Methodology of the study

Each participating neurologist had to complete a data sheet, when they evaluated a patient with migraine (both first and follow-up visits could be included). The data to be collected were as follows: age, gender and referring physician. The baseline characteristics considered were: comorbidities - ischemic cardiac disease, hypertension, (pre) syncope, asthma, nephrolithiasis, gastritis, depression/anxiety, and body mass index (BMI). With respect to current acute treatment, the use of each of the triptans, paracetamol, non-steroidal anti-inflammatory drugs (NSAIDs,) metamizole, opiates and combined drugs was documented. We categorized preventive treatment into five different pharmacological categories, about current and previously used drugs, including: antiepileptic drugs (AEDs) (topiramate (TPM), valproic acid (VPA), pregabalin (PGB), zonisamide (ZNS) and lamotrigine (LMT)), antidepressants (amitriptyline (AMT), serotonin reuptake inhibitors (SSRI), serotonin and norepinephrine reuptake inhibitors (SNRI) (duloxetine, venlafaxine), vortioxetine, opipramol and agomelatine), beta-blockers (BB) and antihypertensive drugs (candesartan and lisinopril), onabotulinumtoxinA (BTX-A) and “others” (calcium Channel Blockers (CCB) (Flunarizine and Cinnarizine), anesthetic blocks, magnesium (Mg) and riboflavin).

 This study was approved by the Hospital de la Princesa´s Ethics Committee (register number: 3477). Patient data were anonymized for data analysis.

### Statistical analyses

We report nominal (categorical) variables as frequencies (percentages) and continuous variables as mean ± standard deviation for normally distributed variables (age and basal headache frequency). We checked normality assumption of quantitative variables through visual methods (Q-Q plots). We assessed statistical significance of comparisons between countries by Pearson’s chi-square test when comparing categorical variables and in the case of having an expected count less than 5 in more than 20 % of cells in the contingency table, we used the Fisher’s exact test. One-way ANOVA was used in order to study group mean differences between countries and patient’s age and headache frequency.

We did not conduct a statistical power calculation prior to the study because the sample size was based on the available data. No missing values were obtained. No adjustment for multiple comparisons were made to the statistical inferences, but exact P values were reported to allow post adjustments, if desired. P values presented are for a two-tailed test and we considered P values < 0.05 statistically significant. All analysis was done using SPSS statistical package, v21.0 for Windows (IBM Corp. Released 2012. Armonk, NY: IBM Corp.)

## Results

### Participants

A total of 734 patients were included but there were statistically significant differences in age and gender between countries. In order to obtain a homogeneous population, we randomly selected age- and gender- matched patients considering the same age-range (20–70 years old) and gender distribution with a similar equally proportional effect between countries. Patients who had not tried any preventive treatment (8.4 %; 62/734) and pregnant patients (or those hoping to be) (3.1 %; 23/734) were not included. Hence, the final sample size considered for the following analysis was 600 patients (30 AU, 100 IT, 80 DE, 45 PL, 60 PT, 85 RUS and 200 ES). 85.4 % (515/600) were females with a mean age of 42.6 ± 11.8 years. Mean headache frequency was 17.5 ± 8.5 days/month and 25.5 % (154/600) of patients had migraines with aura. Chronic migraine (CM) was observed in 63.5 % (381/600) of patients. The median of current and previous preventives used by patient was 3.0. Regarding the main comorbidities that may influence the choice of a migraine preventive treatment, the most prevalent was mood disturbance (anxiety and/or depression), followed by, overweight (BMI ≥ 25), arterial hypertension, gastritis and asthma, in this order. Patients were principally referred by general practitioners and general neurologist. Main demographical data and distribution among countries are shown in Table 1, where significant differences can be seen.

**Table 1:**
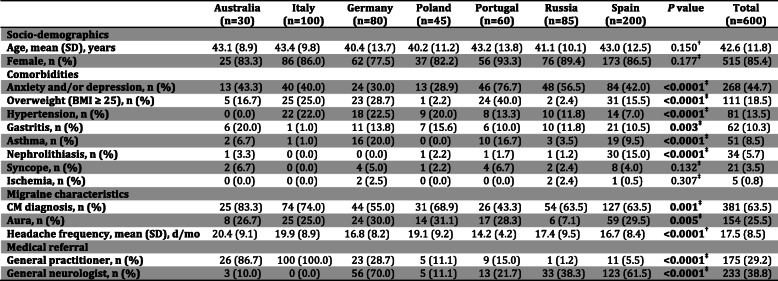
Demographics, migraine characteristics, main comorbidities and medical referral according to each country

Bold font in the P values column indicates statistically significant.

†Significance assessed with chi-square test.

‡Significance assessed with the one-way ANOVA.

*SD* standard deviation; *CM* chronic migraine; *d/mo* days per month.

### Use of migraine acute medication

The most commonly used acute drugs were triptans, especially sumatriptan, followed by NSAIDs and, much less frequently, paracetamol, combined treatments metamizole and opioids (Table 2). Statistically significant differences between countries were found in the use of each acute treatment (Table 2). Triptans were more frequently used in Poland and Russia but in general by at least half of patients in all countries. The use of NSAIDs predominates in countries like Poland and Italy although their use is not as frequent in countries like Australia. In relation to the rest of analgesic medication, their use was drastically reduced compared to the previous two, except for paracetamol in Poland or Australia.

**Table 2:**
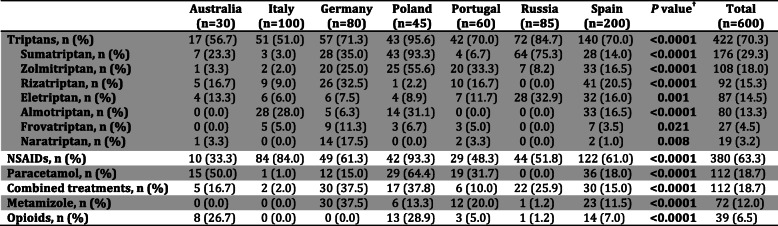
General use (%) of acute treatment according to each country

Bold font in the P values column indicates statistically significant.

†Significance assessed with chi-square test or Fisher’s exact test.

*NSAIDs* non-steroidal anti-inflammatory drug

We set out to study if acute medication prescription in countries was influenced by patient diagnosis or the type of medical referral. For that reason, we only performed the same comparative analysis in CM patients, and thus, homogenized the sample. Once again, triptans (74.0 %, 282/381) and NSAIDs (66.7 %, 254/381) were the most common treatments. Moreover, the same statistically significant differences were observed between countries: while triptans were predominately used in Poland (93.5 %), Russia (84.6 %) and Portugal (74.0 %), NSAIDs were also more frequently used in Poland (96.8 %) and Italy (87.8 %). Regarding medical referral, if we only selected patients referred by general neurologists (38.8 %, 233/600): triptans (73.4 %, 171/233) and NSAIDS (53.6 %, 125/233) were also the standard treatments and non-statistically significant differences were found in triptan prescription between countries (AU: 66.7 %, IT: 79.5 %, DE: 62.5 %, PL: 80.0 %, PT: 76.9 %, RUS: 84.8 %, ES: 74.0 %; *p* = 0.178). However, when we focus the analysis on patients referred by general practitioners (175/600, 29.2 %), differences are observed between countries, both in the use of triptans (AU: 53.8 %, IT: 51.0 %, DE: 91.3 %, PL: 80.0 %, PT: 55.6 %, RUS: 100.0 %, ES: 36.4 %; *p* = 0.012) and NSAIDs (AU: 34.6 %, IT: 84.0 %, DE: 73.9 %, PL: 100.0 %, PT: 55.6 %, RUS: 0.0 %, ES: 63.6 %; *p* < 0.0001).

### Use of preventive migraine medication: pharmacological classes

In this section, we have analyzed the most used treatments considering those that the patient was currently taking as well those that had been prescribed in the past.

**Fig. 1:**
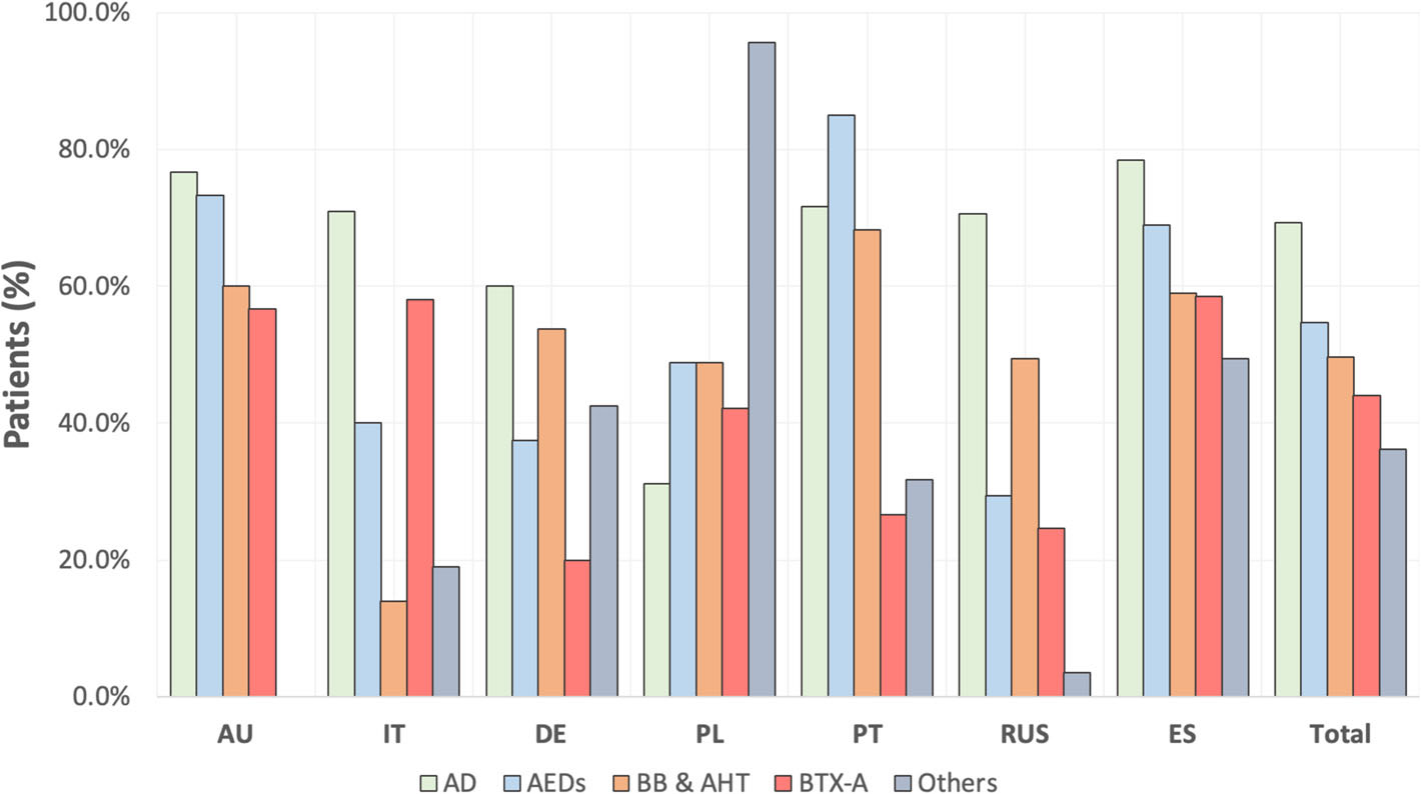
General use (%) of pharmacological classes of preventive treatment according to each country. All treatment classes use between countries resulted statistically significantly different (*p*<0.0001). AU: Australia; IT: Italy; DE: Germany; PL: Poland; PT: Portugal; RUS: Russia; ES: Spain; AD: antidepressants; AEDs: anti-epileptic drugs; BB&AHT: beta-blockers and antihypertensive drugs; BTX-A: Onabotulinumtoxin A.

If we evaluate all the patients as a whole, the order of use would be as follows: antidepressants (69.3 %, 416/600), AEDs (54.7 %, 328/600), beta-blockers and antihypertensive drugs (49.7 %, 298/600), BTX-A (44.0 %, 264/600) and others (36.2 %, 217/600). Statistically significant differences were found between all pharmacological classes and countries (Fig. 1): antidepressants were commonly used in all countries, with the exception of Poland; AEDs were more frequently prescribed in Portugal, Australia and Spain; beta-blockers and antihypertensive drugs were frequently used in all countries except Italy; BTX-A were predominately used in Spain, Italy and Australia and the group of “others” were mainly used in Poland.

**Fig. 2:**
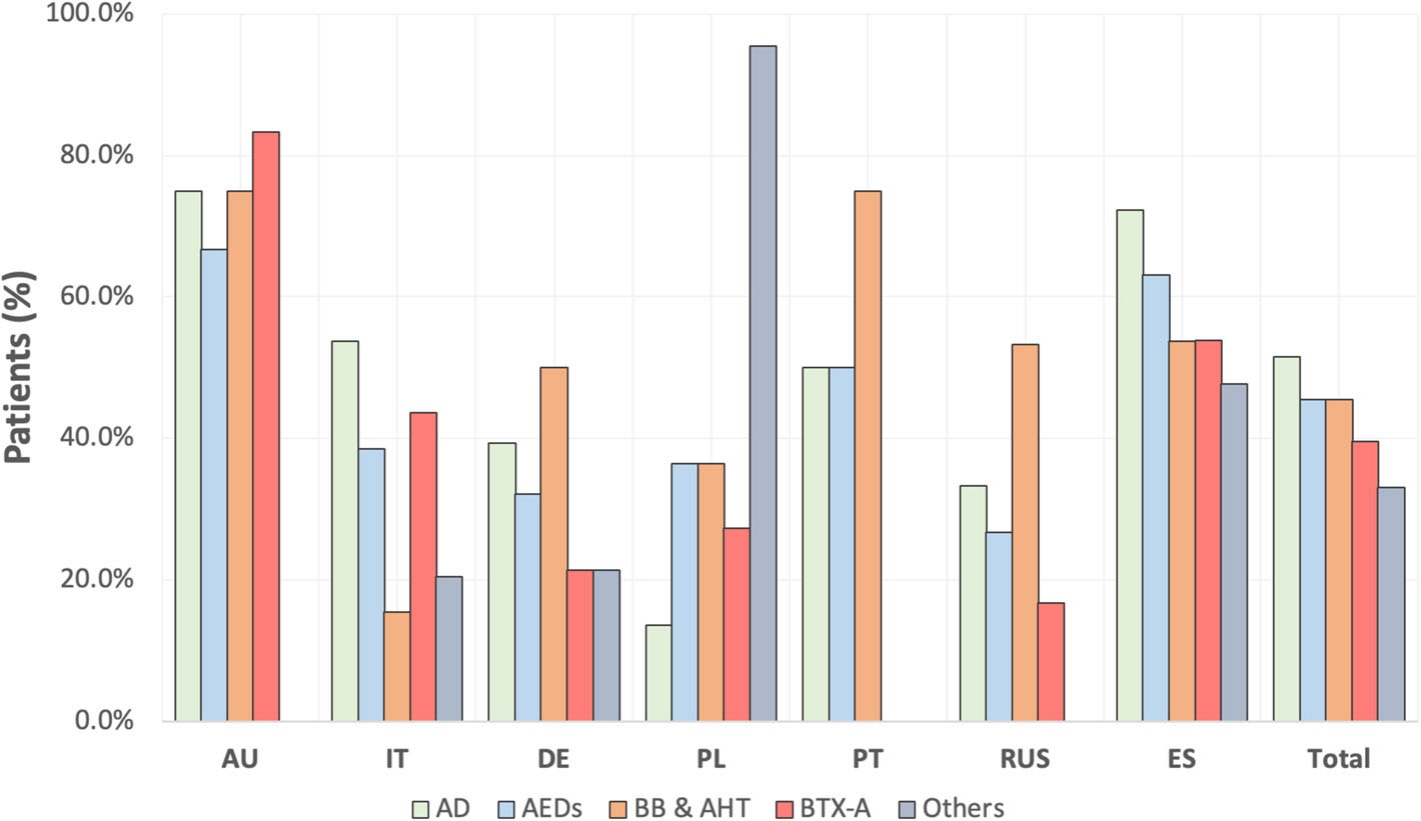
General use (%) of pharmacological classes of preventive treatment according to each country in patients without comorbidities (200/600). All treatment classes use between countries resulted statistically significantly different (*p*<0.0001). AU: Australia; IT: Italy; DE: Germany; PL: Poland; PT: Portugal; RUS: Russia; ES: Spain; AD: antidepressants; AEDs: anti-epileptic drugs; BB&AHT: beta-blockers and antihypertensive drugs; BTX-A: Onabotulinumtoxin A.

Since the selection of the best preventive treatment according to their pharmacological class mainly depends on patient comorbidities, we repeated the same analysis in patients without comorbidities (33.3 %; 200/600) (Fig. 2): antidepressants were most frequently prescribed in Australia, Spain, Italy and Portugal; AEDs were more popular in Australia, Spain and Portugal; beta-blockers and antihypertensive drugs were scarcely used in Italy and Poland; BTX-A was more habitual used in Australia, Spain and Italy and “others” were predominately used in Poland.

### Use of migraine preventive medication: antidepressants

We specifically analyzed antidepressants (AMT, SSRI, SNRI, vortioxetine, opipramol and agomelatine). The most commonly used medications in this pharmacological category were AMT (54.8 %, 329/600), SSRI (30.0 %, 180/600) and SNRI (14.2 %, 85/600). The rest of medications (vortioxetine, opipramol, agomelatine) were only used in 5.2 % (31/600) of participants.

**Fig. 3:**
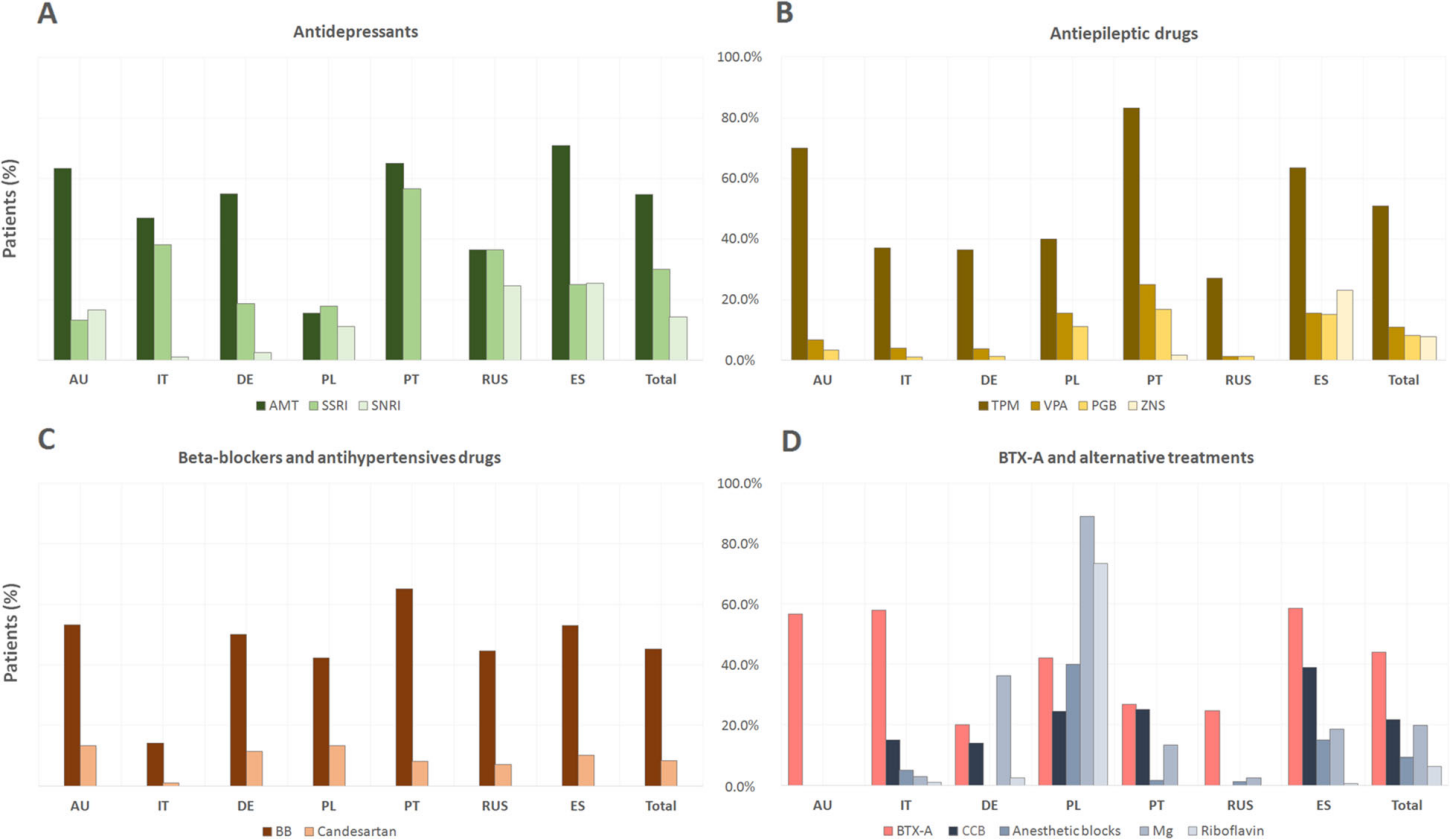
General use (%) of preventive treatment according to each country. AU: Australia; IT: Italy; DE: Germany; PL: Poland; PT: Portugal; RUS: Russia; ES: Spain; AMT: amitriptyline; SSRI: Selective Serotonin Reuptake Inhibitors, SNRI: Serotonine-Norepinephrine Reuptake Inhibitors; TPM: Topiramate; VPA: valproic acid; BTX-A: Onabotulinum Toxin A; PGB: pregabaline; ZNS: zonisamide; BB: beta-blockers; CCB: Calciumchannel blockers.

Statistically significant differences were found in the prescription of these medications between countries (Fig. 3 a): AMT was more frequently used in Spain, Portugal and Australia; SSRIs were more popular in Portugal, Italy and Russia and SNRIs were predominately used in Russia and Spain. The rest are hardly used: vortioxetine (AU: 0.0 %, IT: 2.0 %, DE: 0.0 %, PL: 0.0 %, PT: 1.7 %, RUS: 8.2 %, ES: 1.0 %; *p* = 0.014); Opipramol only in Germany and Poland (AU: 0.0 %, IT: 0.0 %, DE: 11.3 %, PL: 2.2 %, PT: 0.0 %, RUS: 0.0 %, ES: 0.0 %; *p* < 0.0001) and agomelatine in Portugal, Russia and Germany (AU: 0.0 %, IT: 0.0 %, DE: 3.8 %, PL: 0.0 %, PT: 8.3 %, RUS: 1.2 %, ES: 0.0 %; *p* = 0.001).

We found that patients included presented differences in the percentage of anxiety-depression as a comorbidity depending on the country. Hence, we analyzed whether countries prescribed differently these treatments in these patients (44.7 %, 268/600). Once again, the most frequently used treatments were AMT (61.9 %, 166/268), SSRI (58.2 %, 156/268) and SNRI (19.4 %, 52/268). Patients with comorbid anxiety and/or depression were treated differently according to their home country: AMT is mostly used in Spain, Germany and Portugal (AU: 53.8 %, IT: 40.0 %, DE: 79.2 %, PL: 30.8 %, PT: 69.6 %, RUS: 43.8 % and ES: 79.8 %; *p* < 0.0001); SSRIs were predominately used in Italy (AU: 23.1 %, IT: 92.5 %, DE: 45.8 %, PL: 61.5 %, PT: 63.0 %, RUS: 60.4 % and ES: 46.4 %; *p* < 0.0001) and SNRIs were scarcely used in Germany, Portugal and Italy (AU: 23.1 %, IT: 2.5 %, DE: 0.0 %, PL: 30.8 %, PT: 0.0 %, RUS: 6.7 % and ES: 9.7 %; *p* < 0.0001).

**Fig. 4:**
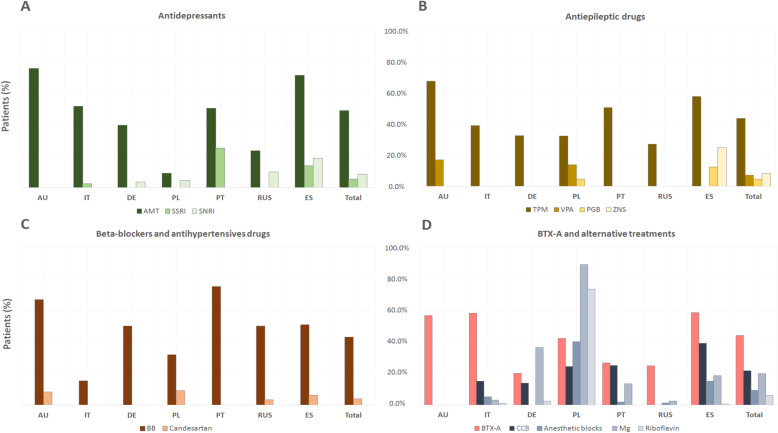
General use (%) of preventive treatment according to each country in patients without comorbidities (200/600). AU: Australia; IT: Italy; DE: Germany; PL: Poland; PT: Portugal; RUS: Russia; ES: Spain; AMT: amitriptyline; SSRI: Selective Serotonin Reuptake Inhibitors, SNRI: Serotonine-Norepinephrine Reuptake Inhibitors; TPM: Topiramate; VPA: valproic acid; BTX-A: Onabotulinum Toxin A; PGB: pregabaline; ZNS: zonisamide; BB: beta-blockers; CCB: Calciumchannel blockers.

Finally, we studied the prescription of antidepressants in patients without comorbidities (Fig. 4 a) between different countries: AMT was the main medication used and their prescription remained statistically significantly different between countries.

### Use of migraine preventive medication: antiepileptic drugs (AEDs)

In this section, we particularly analyzed the different neuromodulator treatments. The most widely use neuromodulator treatment used was TPM (50.8 %, 305/600) followed by VPA (10.8 %, 65/600), PGB (8.2 %, 49/600), ZNS (7.8 %, 47/600) and LMT (2.8 %, 17/600). Statistically significant differences between AEDs used were also found between countries (Fig. 3 b): TPM was predominately used in Portugal, Australia and Spain; VPA and PGB were more popular in Portugal, Spain and Poland and ZNS was practically only used in Spain. No statistically significant differences were found in the use of LMT between countries.

Finally, we studied the prescription of AEDs between countries in patients without comorbidities (Fig. 4 b): TPM was the main medication used and their prescription remained statistically significantly different between countries.

### Use of migraine preventive medication: beta-blockers and antihypertensive drugs

Here we studied the distinctive use of BB, candesartan and lisinopril in different countries. In this pharmacological subgroup (Fig. 3 c), BBs were the most popular treatment (45.3 %, 272/600) followed by candesartan (8.5 %, 51/600) and lisinopril (2.7 %, 16/600). BBs and candesartan were commonly used in all countries, with the exception of Italy. Lisinopril was hardly used except in Spain (AU: 0.0 %, IT: 0.0 %, DE: 1.3 %, PL: 0.0 %, PT: 0.0 %, RUS: 0.0 %, ES: 7.5 %; *p* < 0.0001).

The prescription of beta-blockers and antihypertensive drugs may vary in patients with cardiovascular risk. In our study, we wanted to analyse the differential use of this pharmacological subgroup in patients with overweight, hypertension and/or cardiac ischemia (26.3 %, 158/600) between countries. In this subgroup, most frequently treatments were also BBs (51.3 %, 81/158) and candesartan (13.9 %, 22/158). While no differences were found in candesartan prescription between countries (*p* = 0.113), the similar statistically significant use was found with BBs (AU: 40.0 %, IT: 13.9 %, DE: 65.5 %, PL: 60.0 %, PT: 65.4 %, RUS: 63.6 % and ES: 61.0 %; *p* < 0.0001).

Finally, the prescription of BBs in patients without comorbidities was also statistically significantly different between countries (Fig. 4 c). No differences were found in the use of candesartan and lisinopril between countries in this subset of patients.

### Use of migraine preventive medication: other treatments

In this group, the most commonly used treatments were CCB (21.7 %, 130/600) followed by Mg (19.8 %, 119/600), anaesthetic blocks (9.2 %, 55/600) and riboflavin (6.2 %, 37/600). In relation to other countries (Fig. 3 d), CCB has a statistically significant higher use in Spain but Mg, anaesthetic blocks and riboflavin were predominately used in Poland.

The same proportional use between countries was observed in patients without comorbidities (Fig. 4 d).

### Migraine treatment use according to patient comorbidity

Statistically significant differences were found in the global set of all patients in the use of preventive treatments considering the patient´s previous pathology. Thus, we finally studied the differential prescription of pharmacological subgroups and the presence/absence of frequent comorbidities in our cohort (presence ≥ 10 %). In this context, patients with presence of anxiety and/or depression were statistically significantly more frequently prescribed with antidepressants (absence: 53.3 % vs. presence: 89.2 %; *p* < 0.0001), AEDs (absence: 49.1 % vs. presence: 61.6 %; *p* = 0.002) and BTX-A (absence: 38.3 % vs. presence: 51.1 %; *p* = 0.003). Popular pharmacological categories in overweight patients were antidepressants (absence: 67.5 % vs. presence: 77.5 %; p = 0.041) and AEDs (absence: 52.1 % vs. presence: 65.8 %; *p* = 0.011). Specifically, the use of TPM is higher 60.4 % (67/111 subgroup of overweight patients) vs. 50.8 % (305/600 total sample) and the use of CCB is slightly lower 20.7 % (23/111) vs. 21 % (130/600), and this is even less if both comorbidities are present, being overweight and experiencing mood alteration (16.1 % (9/56) vs. 21 %). Surprisingly, the use of VPA in overweight patients is slightly higher 11.7 % (13/111) vs. 10.8 % (65/600). Regarding hypertension, patients were more commonly treated with beta-blockers/antihypertensive drugs (absence: 47.6 % vs. presence: 63.0 %; *p* = 0.012) and BTX-A (absence: 42.2 % vs. presence: 55.6 %; *p* = 0.030). The use of BB was lower in patients with asthma (absence: 46.1 vs. presence: 37.3 % *p* = 0.243). Finally, patients with gastritis were more commonly prescribed alternative treatments (absence: 34.8 % vs. presence: 48.4 %; *p* = 0.037).

## Discussion

There are multiple reviews on the best preventive treatments for patients with migraine [7–11], as well as European and American recommendations [3, 4, 12] and national guidelines in each country [5, 6, 13, 14], however there is no consensus on which the drug of first choice is, due to the limitations of comparative studies carried out to date, with few head to head comparisons [15, 16], where there is only one study which also includes non-oral treatments [17].

This is the first international multicentre study with the aim of comparing whether there are differences in the use of acute and preventive migraine treatments when analyzing the drugs prescribed in seven countries.

It can be seen that there is no single first-line drug of choice as preventive treatment, and significant differences can be observed between countries and there is a wide arsenal of drugs used, opting for one or the other according to the patient´s profile and their comorbidities, but there are also differences in relation to the nationality of the prescribing physician.

In our study, we have analyzed a total of 734 patients from seven different countries (Australia, Italy, Germany, Poland, Portugal, Russia and Spain), from specialized headache units, which implies a more complex patient profile, often with a long pharmacological history. The mean number of days of pain per month was almost 20, with a high percentage of drugs tested by each patient, over all in Spain, Portugal and Poland.

In relation to acute treatment, given the patient’s profile and being monitored in a specialized unit, it is not surprising to find that the percentage of use of triptans is generally high in all countries. Sumatriptan is the most widely used of these, possibly because it has the longest therapeutic course and naratriptan and frovatriptan was the least used, perhaps because these are usually considered to be more tolerable but a bit slower and with longer half-lives [18]. However, there are also differences between countries; sumatriptan is used much more in countries like Poland (93.3 %) or Russia (75.3 %) compared to others such as Italy, which only accounts for 3 % or Portugal 6.4 %. Nevertheless, when we separate this aspect in relation to the referring physician, it becomes clear that this difference is possibly due more to whether the patient had been referred by primary care or general neurology, than actually to nationality. NSAIDs are also widely used, in contrast to other analgesics such as paracetamol, metamizole, opioids and combined, in line with international recommendations [19, 20].

Regarding migraine preventive treatment, the most commonly used drugs were antidepressants (69.3 %), AEDs (54.7 %) and beta-blockers (49.7 %), in this order. However, at this point we found significant differences between countries.

Firstly, in relation to the use of antidepressants, AMT was used in all countries in more than 1/3 of patients (especially in Spain, Portugal and Australia), except in Poland (16 %). In relation to the other antidepressants, the next ones in use are the SSRIs, especially in countries such as Portugal, Italy or Russia. SNRIs are less used in general, with Russia and Spain as exceptions. AMT was also the most used drug, both in the subgroup of patients with anxiety and/or depression, whereas in countries like Portugal it represents a very high percentage, and in the subgroup without comorbidities.

Secondly, the most widely used neuromodulator treatment was TPM, also in patients without comorbidities, however countries like Russia use it significantly less, approximately in 1/4 patients, compared to others such as Portugal or Australia, where it has been prescribed in 3/4 or Spain in 2/3. In a much smaller percentage, VPA and PGB were more popular in Portugal, Spain and Poland.

BB were the most used preventive treatment within the group of antihypertensives. BB and candesartan were commonly prescribed in all countries, with the exception of Italy.

BTX-A, a first-line drug together with TPM in chronic migraine [21], is widely used, also considering that they are patients in specialized units, especially in Spain, Italy and Australia.

In relation to anaesthetic blocks, their use is much less common, except in Poland (40 %), where the use of nutraceuticals also predominates. Here, almost all patients have tried Riboflavine or Mg (the latter is used more by the rest of the countries except for Australia, Italy and Russia).

In the use of CCB, important differences appear, such as its use in Spain of almost 40 % vs. in countries such as Australia or Russia, where no prescriptions were made.

Drugs like pregabalin, LMT, vortioxetine, agomelatine are hardly used. There are drugs such as ZNS and lisinopril, which were prescribed almost exclusively in Spain or Opipramol in Germany. Possibly due to low scientific evidence [22, 23] except in the case of lisinopril [24].

The findings in relation to the use of preventive treatments and patient comorbidity are consistent with our usual clinical practice. Therefore, in those patients with previous cardiac ischemia or who have hypertension, BB or antihypertensives such as candesartan for their dual effect are commonly used; and, we avoid them in patients with asthma in the case of non-selective BB [25]. Another key point, looking for synergistic effects is also mood comorbidity. In these patients we use drugs with double effect, such as AMT, SSRI, SNRI or drugs that do not worsen it, such as antihypertensives and we reject CCB [26], which also lead to weight gain. Some do the opposite such as TPM, while others have no effect on weight such as BTX-A and this is also observed in the choice of preventive treatment [27].

Nevertheless, when only analyzing patients without comorbidities - in order not to affect the choice of treatment - significant differences persist in all pharmacological groups.

This study presents a number of considerations that must be taken into account. The main limitation of our study is that for each country only one or two centres participated, so it must not be inferred that it is a representation of the global prescribing trends of each country, but rather it is based on the opinion of neurologists of different nationalities. However, in relation to this point, it is important to consider that all participating physicians work in specific headache units and all of them have been trained in line with the guidelines of each country and they follow their national recommendations. It should also be noted that the number of patients included in some countries is low in relation to others, which means that perhaps if the size of patients analyzed were increased, the differences could be greater. We have not divided the uses in episodic or chronic migraine due to the fact that all drugs prescribed to the patient are analyzed, where it is not possible to know whether a drug was prescribed in a period with a lower or higher frequency of attacks. Moreover, in the list of preventive treatments taken by the patient, it was not indicated who had made the prescription and it is possible that some of the first treatments used were not prescribed in the specialized headache centre, rather by the primary care physician or the general neurologist, although these patients usually have an early referral due to their complexity and, likewise, there is an the overall trend in the country. Lastly, with regard to treatment with anti-CGRP monoclonal antibodies, the results are not included because in many of the countries included in the study at the time when this study was carried out, they were not approved by their National Healthcare Service.

## Conclusions

In the process of choosing the most appropriate acute or preventive migraine treatment, comorbidities, the patient’s opinion [28], and of course, that of the neurologist must always be assessed, but as we have observed in this first international study, this may differ depending on the country where the treatment is prescribed.

In fact, the European guidelines to dictate the new monoclonal treatments against CGRP [29] indicate that at least two oral treatments must be prescribed (and BTX-A in the case of chronic migraine), however, it is not specified which should be the two first options.

This investigation shows the need for a larger multicentre study to verify these results and to continue with comparative clinical trials between the first line treatments, in order to create global international algorithms that guarantee the best therapeutic option for our patients.

## Data Availability

Study material and supplementary material are available upon request to the corresponding author.

## Abbreviations

AMT: Amitriptyline
AU: Australia
BB: Beta-Blokers
BMI: Body mass index
BTX-A: Onabotulinumtoxin A
CCB: calcium channel blockers
CGRP: calcitonin gene related peptide
ES: Spain
DE: Germany
IT: Italy
LMT: Lamotrigine
Mg: Magnesium
PL: Poland
PT: Portugal
PGB: Pregabalin
RUS: Russia
SNRI: Serotonin and Norepinephrine reuptake inhibitors
SSRI: Serotonin reuptake inhibitors
TPM: Topiramate
VPA: Valproic acid
ZNS: Zonisamide

## References

[1] Steiner TJ, Stovner LJ, Jensen R (2020). Migraine remains second among the worlds-s causes of disability, and first among young women: findings from GBD2019. J Headache Pain.

[2] Lipton RB, Bigal ME, Diamond M, Freitag F, Reed ML, Stewart WF, AMPP Advisory Group (2007). Migraine prevalence, disease burden, and the need for preventive therapy. Neurology.

[3] Loder E, Burch R, Rizzoli P (2012). The 2012 AHS/AAN guidelines for prevention of episodic migraine: a summary and comparison with other recent clinical practice guidelines. Headache.

[4] Evers S, Afra J, Frese A (2009). EFNS guideline on the drug treatment of migraine – revised report of an EFNS task force. Eur J Neurol.

[5] Diener H-C, Holle-Lee D, Nägel S et al. Treatment of migraine attacks and prevention of migraine: Guidelines by the German Migraine and Headache Society and the German Society of Neurology. Clinical and Translational Neuroscience. 2019

[6] Ezpeleta d, Pozo-Rosich P. Guías diagnósticas y terapéuticas de la Sociedad Española de Neurología. Guía oficial de práctica clínica en Cefaleas. Ed. Luzán 5, Madrid. 2015

[7] Headache Classification Committee of the International Headache Society (HIS). The International Classification of Headache Disorders, 3rd edition. Cephalalgia 2018; 38: 1-211 10.1177/0333102417738202 29368949

[8] Parikh Sk, Silverstein SD (2019). Preventive treatment for episodic migraine. Neurol Clin.

[9] Schwedt TJ (2018). Preventive therapy of migraine. Continuum.

[10] Rizzoli P (2014). Preventive pharmacotherapy in migraine. Headache.

[11] Lipton R, Silberstein S (2015) Episodic and chronic migraine headache: Episodic and chronic migraine headache: breaking down barriers to optimal treatment and prevention. Headache 55:103–122 10.1111/head.12505_2 25662743

[12] The American Headache Society (2019). Position Statement on Integrating new migraine treatments into clinical practice. Headache.

[13] Sarchielli P, Granella F, Prudenzano MP, Pini LA, Guidetti V, Bono G, Pinessi L, Alessandri M, Antonaci F, Fanciullacci M, Ferrari A, Guazzelli M, Nappi G, Sances G, Sandrini G, Savi L, Tassorelli C, Zanchin G. Italian guidelines for primary headaches: 2012 revised version. J Headache Pain 2012; Suppl2: S31-70 10.1007/s10194-012-0437-6 PMC3350623 22581120

[14] Lantéri-Minet M, Demarquay G, Alchaar H, Bonnin J, Cornet P, Douay X, Dousset V, Géraud G, Guillouf V, Navez M, Radat F, Radenne S, Revol A, Valade D, Donnet A (2014). Management of chronic daily headache in migraine patients: medication overuse headache and chronic migraine. French guidelines. Rev Neurol (Paris).

[15] Jackson J, Cogbill E, Santana-Davila R, Eldredge C, Collier W, Gradall A, Sehgal N, Kuester J (2015). A comparative effectiveness meta-analysis of drugs for prophylaxis of migraine. PloS One.

[16] Ashtari F, Shaygannejad V, Akbari M (2008). A double-blind, randomized trial of low-dose topiramate vs propanolol in migraine prophylaxis. Acta Neurol Scand.

[17] Rothrock JF, Adams AM, Lipton RB, Silberstein SD, Jo E, Zhao X, Blumenfeld AM (2019). FORWARD Study investigative group. FORWARD Study: Evaluating the Comparative Effectiveness of OnabotulinumtoxinA and Topiramate for Headache Prevention in Adults with Chronic Migraine. Headache.

[18] Ferrari MD, Roon KI, Lipton RB, Goadsby PJ (2001). Oral triptans (serotonin 5-HT (1b/1d) agonist) in acute migraine treatment: a meta-analysis of 53 trials. Lancet.

[19] Marmura MJ, Silberstein S, Schwedt T (2015). The acute treatment of migraine in adults: the american headache society evidence assessment of migraine pharmacotherapies. Headache.

[20] Cameron C, Kelly S, Hsieh SC, Murphy M, Chen L, Kotb A, Peterson J, Coyle D, Skidmore B, Gomes T, Clifford T, Wells G (2015). Triptans in the acute theatment of migraine: A systematic Review and Network Meta-analysis. Headache.

[21] Aurora SK, Dodick DW, Turkel CC, DeGryse RE, Silberstein SD, Lipton RB, Diener HC, Brin MF (2010). PREEMPT 1 Chronic Migraine Study Group. Cephalalgia.

[22] Pascual-Gómez J, García-Naya M, Leira R, Mateos V, Alvaro-Gonzalez C, Hernando I (2010). Et al. Zonisamide in the preventive treatment of refractory migraine. Rev Neurol.

[23] Jacobs H (1972). A trial of opipramol in the treatment of migraine. J Neurol Neurosurg Psychiatry.

[24] Schrader H, Stovner LJ, Helde G et al. Prophylactic treatment of migraine with an angiotensin converting enzyme inhibitor (Lisinopril): randomised, placebo controlled, crossover study. BMJ 322: 19–22 10.1136/bmj.322.7277.19 PMC26600 11141144

[25] Kuipers E, Wensing M, De Smet, Teicher M (2018). Considerations of prescribers and pharmacists for the use of non-selective B-Blockers in asthma and COPD patients. J Eval Clin Pract.

[26] Lugaresi A, Montagna P, Gallasi R, Lugaresi E. Extrapyramidal syndrome and depression induced by flunarizine. Eur Neurol 188; 28: 208–11 10.1159/000116268 3416889

[27] Taylor F (2008). Weight change associated with the use of migraine-preventive medications. Clin Ther.

[28] Dekker F, Knuistingh A, Andriesse B, Kernick D, Reis R, Ferrari M et al (2012) Prophylactic treatment of migraine; the patient´s view, a qualitative study. BMC Fam Pract 9:13:13 10.1186/1471-2296-13-13 PMC3359207 22405186

[29] Sacco S, Bendtsen L, Ashina M, Reuter U, Terwindt G, Mitsikostas DD, Martelletti P (2019). European headache federation guideline on the use of monoclonal antibodies acting on the calcitonin gene related peptide or its receptor for migraine prevention. J Headache Pain.

